# Effect of socio-cultural factors on spontaneous abortion in Burdur, Turkey: A population based case-control study

**DOI:** 10.12669/pjms.325.10078

**Published:** 2016

**Authors:** Binali Catak, Can Oner, Sevinc Sutlu, Selcuk Kilinc

**Affiliations:** 1 Binali Catak, MD. Assistant Professor, Department of Public Health, Kafkas University, School of Medicine, Kars, Turkey; 2 Can Oner, MD. Assistant Professor, Department of Family Medicine, Dr. Lutfi Kirdar Kartal Education and Training Hospital, Istanbul, Turkey; 3 Sevinc Sutlu, MD. Burdur Public Health Directory, Turkey; 4 Selcuk Kilinc, MD. Burdur Public Health Directory, Turkey

**Keywords:** Abortion, Factors, Case Control Studies, Miscarriage, Socioeconomic, Spontaneous Abortion

## Abstract

**Objective:**

To determine the sociocultural factors that have effect on spontaneous abortion in Burdur, Turkey.

**Methods:**

Study was designed as case-control study. The case group consist of 257 women whose pregnancies ended with spontaneous abortion. The control group consisted of 514 women whose pregnancy continued since 22 weeks and more during the study. Chi-square, and backward LR logistic regression were utilized in analyses.

**Results:**

In multifactorial-analyses it was determined that four factors (educational status of women, employment status of women, exposure to physical violence and non-receipt of ANC) created independent risk on spontaneous abortions.

**Conclusions:**

Pregnant women with these risk factors should be followed up more frequently and in a more qualified way in primary and secondary and tertiary health institutions.

## INTRODUCTION

Spontaneous abortion is one of the most common pregnancy related adverse outcomes, it is defined as premature loss of fetus and fetal attachments -completely or partially from the uterus- up to 20 weeks of pregnancy. It is estimated that 8% of pregnancies ended in clinically recognized spontaneous abortion and this rate is estimated up to one third in clinically unrecognized pregnancies.^1^ This rates reach up to 14% in national data.^2^ Due to its high frequency and identification of a small potential for prevention, spontaneous abortion has significant impact on public health.^3^

There are many risk factors related with spontaneous abortion especially fetal malformations and chromosomal abnormalities, chronic diseases of mothers, uterine disorders, immunological factors and infections.^3^ On the other hand several modifiable risk factors for spontaneous abortion was also identified: Older maternal and paternal age, obesity, smoking, alcohol and caffeine consumption.^4^ It has been shown in some studies that sociocultural factors like educational status, employment, place of residence and social classes play role in spontaneous abortion. Women with lower educational levels, unemployed women, women in lower social classes have increased risk of spontaneous abortion.^3^

Most of the studies about spontaneous abortions have often been conducted based on hospital databases. The important limitation of these studies is including women only who are admitted to hospitals. The elucidation of the relationship between spontaneous abortion and social factors may help us to indicate preventable factors like environmental and behavioral factors etc. It is possible to follow up pregnant women with determined socio-cultural risk factors more frequently and in a more qualified way for spontaneous abortion. But there are a few population-based studies investigating factors that affect spontaneous abortions in the literature.^5^ The aim of this study was to determine the relationship between social factors and spontaneous abortions.

## METHODS

The study was carried out in the province of Burdur, Turkey between January 1, 2011 - December 31, 2011. Burdur is a city located at Mediterranean region of Turkey. Population of the province was 254411 in the study period and 54827 of them were women at reproductive age (15-49 years). There are 78 family health units. Approximately 3262 people benefit from each family health unit. A family health unit is a basic unit for primary health services composed on one physician and one family health midwife providing services for a maximum of 4,000 people.

A family unit is obliged to furnish primary curative, preventive and rehabilitative health services to people who are registered to them and notify the Health Care Department incident to such given services. One of the preventive health services provided by family health unit is the follow-up of pregnant women. Family physicians identify and follow up pregnant women according to national guidelines and inform the Public Health Directorate as to the termination means of pregnancy (spontaneous abortion, stillbirth, live births).

The study group of this study consisted of all women whose pregnancies were clinically diagnosed and included to pregnancy follow-up by a physician but their pregnancies ended with spontaneous abortion between the study period (n=257). The control group consisted of women who were pregnant during the same period and whose pregnancy continued since 22 weeks and more during the study. A sample was not selected in the study group. It was decided to take two controls for every one study and the number of women to be included to the control group was calculated as 514. Matching factors, for the control group, were taken as receipt of service from the same family health care unit and living in the same area (alley or street or neighborhood) with the study group. Pregnant women lists of all family physicians were created beforehand based on the matching factor for the selection of the control group. Subsequently 514 pregnant women were identified by virtue of the random numbers table from these created lists. Two hundred and forty-nine (96.9%) of study group and 503 (97.9%) of controls have been approached. The most important reason of the failure in approaching women was address change of the participants. The study was approved by Burdur Public Health Directorate (27/04/2012-538) and all participants gave verbal informed consent.

We have prepared a data collection form including questions about the women’s socio-demographic and -economic features, obstetrical history, health care properties and spousal violence. The data were collected by midwives working in the public health centers. Midwives were given a five-hour training program including important issues during the data collection phase (purpose of the study, questions, data collection methods, etc.). To minimize the possible mistakes arising from forgetting, midwives met women in study group within two weeks following spontaneous abortion. The data of the study were collected after obtaining necessary permissions from the Public Health Directorate and women’s verbal consents.

Data were analyzed by SPSS 20.0 package program. Chi-squared and backward logistic regression tests were utilized in analyses. The odds ratio, and the confidence intervals (CI) were calculated. To determine factors affecting spontaneous abortions, the independent variables were analyzed with the chi-squared test. The statistically significant (p <0.05) variables in chi-squared test were integrated into the backward logistic regression analysis model.

## RESULTS

The study included 752 women (249 cases/503 controls). Table-I shows the distribution of demographic features on study and control groups. It can be seen that there is a statistically significant difference between the study and control groups in terms of age of the women (p=0.048), total number of pregnancies (p=0.006), educational status of women (p=0.001), educational status of their husbands (p=0.048), employment status of women (p=0.004) and employment status of their husbands (p=0.038).

**Table-I:** Sociodemographic features of both groups.

*Sociodemographic features*		Case n (%)*	Control n (%)*	*χ^2^*	*P*
Age of women	≤19	16 (6.4)	51 (10.1)	6.092	0.048
	20–34	196 (78.7)	402 (79.9)		
	≥35	37 (14.9)	50 (9.9)		
Marriage age	≤19	102 (41.0)	122 (44.1)	1.800	0.406
	20–29	136 (54.6)	267 (53.1)		
	≥30	11 (4.4)	14 (2.8)		
Age of first pregnancy	≤19	74 (29.7)	169 (33.6)	1.244	0.537
	20–29	160 (64.3)	308 (61.2)		
	≥30	15 (6.0)	26 (5.2)		
Menarche age	≤13	149 (59.8)	278 (55.3)	1.418	0.234
	≥14	100 (40.2)	225 (44.7)		
Total pregnancy	1	61 (24.5)	175 (34.8)	10.274	0.006
	2–3	137 (55.0)	257 (51.1)		
	≥4	51 (20.5)	71 (14.1)		
Place of residence	Village	70 (28.1)	134 (26.6)	0.183	0.669
	City	179 (71.9)	369 (73.4)		
Family type	Extended Family	41 (16.5)	96 (19.1)	0.767	0.394
	Nuclear Family	208 (83.5)	407 (80.9)		
Formal marriage**	Informal	4 (1.6)	8 (1.6)	0.001	0.979
	Formal	243 (96.4)	495 (98.4)		
Kin marriage	Present	19 (7.6)	36 (7.2)	0.055	0.814
	Not	230 (92.4)	467 (92.8)		
Count of household (person #)	4 and below	213 (85.5)	438 (87.3)	0.421	0.516
	5 and above	36 (14.5)	64 (12.7)		
Education level of woman	5 years and below	113 (45.4)	146 (29.0)	19.732	0.001
	6 years and above	136 (54.6)	357 (71.0)		
Education level of man	5 years and below	74 (29.7)	116 (23.1)	3.909	0.048
	6 years and above	175 (70.3)	387 (76.9)		
Working status of woman	Employed	47 (18.9)	56 (11.1)	8.446	0.004
	Unemployed	202 (81.1)	447 (88.9)		
Working status of man	Employed	225 (90.4)	475 (94.4)	4.290	0.038
	Unemployed	24 (9.6)	28 (5.6)		
Health insurance of woman	Non	9 (3.6)	13 (2.6)	0.622	0.430
	Present	240 (96.4)	490 (97.4)		
Total	249 (100.0)	503 (100.0)			

* column percentage,

** 2 missing data from study group,

# 1 missing data from control group.

When evaluated in terms of health feature there is a statistically significant difference between the study and control groups in terms of women’s desire for pregnancy (p=0.043), desire for women’s pregnancy by her spouse (p=0.014) and receipt of Antenatal care (ANC) by women in the first 14 weeks (p=0.001) (Table-II).

**Table-II:** Health features of women.

*Health features*		Study n (%)*	Control n (%)*	*χ^2^*	*P*
Unintended pregnancy (woman)	Yes	25 (10.0)	30 (6.0)	4.082	0.043
	No	224 (90.0)	473 (94.0)		
Unintended pregnancy (man)**	Yes	22 (8.9)	22 (4.4)	6.091	0.014
	No	226 (91.1)	481 (95.6)		
Pregnancy type	Normal	233 (93.6)	473 (94.0)	0.062	0.804
	Assisted techniques	16 (6.4)	30 (6.0)		
Diagnostic procedure of pregnancy	Urine samples	74 (29.7)	163 (32.4)	1.413	0.493
	Blood samples	153 (61.4)	287 (57.1)		
	USG	22 (8.8)	53 (10.5)		
Antenatal care within first 14 weeks	No	42 (16.9)	45 (8.9)	10.215	0.001
	Yes	207 (83.1)	458 (91.1)		
Contraception method before pregnancy	Any	109 (43.8)	231 (45.9)	1.133	0.568
	Modern methods	79 (31.7)	166 (33.0)		
	Conventional methods	61 (24.5)	106 (21.1)		
Rh incompatibility≠	Yes	23 (9.5)	47 (9.4)	0.004	0.950
	No	218 (90.5)	453 (90.6)		
Chronic disease of woman	Yes	18 (7.2)	41 (8.2)	0.196	0.658
	No	231 (92.8)	462 (91.8)		
Genital system operation history	Yes	8 (3.2)	30 (6.0)	2.628	0.105
	No	241 (96.8)	473 (94.0)		
Genital infection history	Yes	56 (22.5)	102 (20.3)	0.491	0.484
	No	193 (77.5)	401 (79.7)		
Drug use	Yes	27 (10.8)	43 (8.5)	1.039	0.308
	No	222 (89.2)	460 (91.5)		
Menstrual irregularity	Yes	24 (9.6)	67 (13.3)	2.122	0.145
	No	225 (90.4)	436 (86.7)		
Total		249 (100.0)	503 (100.0)		

* column percentage,

** 1 missing data from study group,

≠ 8 missing data form study and 3 missing data from control group.

There is a statistically significant difference in the study group in terms of exposure to physical violence between those who had spontaneous abortion and those who had not spontaneous abortion (P=0.001) (Table-III).

**Table-III:** Health behaviors of couples.

*Health behaviors and domestic violence*		Study n (%)*	Control n (%)*	*χ^2^*	*P*
Smoking (women)	Yes	25 (10.0)	57 (11.3)	0.286	0.593
	No	224 (90.0)	446 (88.7)		
Passive smoking (Women)	Yes	82 (32.9)	139 (27.6)	2.252	0.133
	No	167 (67.1)	364 (72.4)		
Alcohol (Women)	Yes	3 (1.2)	3 (0.6)	0.779	0.377
	No	246 (98.8)	500 (99.4)		
Coffee (Women)	Yes	43 (17.3)	71 (14.1)	1.288	0.256
	No	206 (82.7)	432 (85.9)		
Smoking (men)	Yes	136 (54.6)	287 (57.1)	0.403	0.526
	No	113 (45.4)	216 (42.9)		
Alcohol (men)	Yes	83 (33.3)	171 (34.0)	0.033	0.857
	No	166 (66.7)	332 (66.0)		
Verbal violence **	Yes	63 (25.3)	112 (22.3)	0.833	0.361
	No	186 (74.7)	390 (77.7)		
Sexual violence**	Yes	28 (11.3)	37 (7.4)	3.222	0.073
	No	220 (88.7)	465 (92.6)		
Physical violence	Yes	38 (15.3)	37 (7.4)	11.592	0.001
	No	211 (84.7)	466 (92.6)		
Total		249 (100.0)	503 (100.0)		

* column percentage,

** 1 missing data from both study and control group.

Logistic regression analysis results table, including the factors affecting spontaneous abortion is shown in Table-IV. According to this, spontaneous abortion is seen 2.3 times more in women who have received education for 5 years and less compared to women who have received education for 6 years and over (CI: 1.7 to 3.3); 2.1 times more in women who have received ANC within 14 weeks compared to women who have not received ANC within 14 weeks (CI: 1.3 to 3.4); 2.2 times more in working women compared to women who are not working (CI: 1.4 to 3.5) and 2.0 times more in women who experience physical violence compared to women who do not experience physical violence (CI: 1.2 to 3.2).

**Table-IV:** Result of Logistic regression analyses.

*Independent variables*		*Odds Ratio*	*%95 CI*
Education level of women	5 years or below	2.3	1.7-3.3
	6 years or above	1 (reference)	
Working status of women	Employed	2.2	1.4-3.5
	Unemployed	1(reference)	
Physical violence	Yes	2.0	1.2-3.4
	No	1(reference)	
Use of Antenatal care services	No	2.1	1.3-3.4
	Yes	1(reference)	

## DISCUSSION

Several independent variables possibly affecting spontaneous abortion were examined in our study. As a result four variables were defined as risk factors for spontaneous abortion: Educational (OR: 2.3; CI=1.7-3.3) and employment status of women (OR:2.2; CI=1.4-3.5), physical violence (OR:2.0; CI1.2-3.4) and access to antenatal care within 14 week of pregnancy (OR:2.1; CI:1.3-3.4).

We found out in this study that spontaneous abortion is 2.3 times more in women with ≤ 5 years of education compared to women who have ≥6 years. In a recent study socioeconomic position and the risk of spontaneous abortion was investigated. It was reported that women with <10 years of education has 1.19 times (CI:1.05-1.35) more elevated risk of spontaneous abortion compared.-with women with >12 years of education.^3^ On the other hand, there are also studies indicating no relationship between spontaneous abortion and educational level.^6^,^7^ We believe that low educational level is effective on the risk of spontaneous abortion due to late recognition of danger signs during pregnancy and late admission to the hospital.^8^ Women’s educational level is also directly related with household wealth and empowers women in household decision making.^9^

The relationship between employment statuses of women with miscarriage is not obvious in literature. In a study from Japan, it was reported that spontaneous abortion is seen 1.65 times more in employed mothers.^10^ But in another study it was shown that unemployed women had the same risk of spontaneous abortion as the employed women.^3^ Adel et al noted an increased risk of miscarriage in unemployed group compared employed ones.^7^ This difference is due to the employment status of women in different country and difference of study methodology. We found in this study that spontaneous abortion is seen 2.2 times more in employed women.

We thought that employment status of women plays an important role in miscarriage. In a recent study in European Union it was demonstrated that 16% of employed women works in shifts, 13% work at night and 16% work more than 40 hours a week. Moreover 15% women works in tiring or painful positions and 23% of women carry or move heavy loads^11^. The national data shows that significant proportion of women work in agricultural and private sectors (especially textile and service sectors) and mainly informally (black economy).^12^ The link between miscarriage and employment is due to working conditions, ergonomics and its effect on social class. Miscarriage in employed women are thought to be related with prolonged work, working in different shifts and night work, heavy lifting, wrong posture during work and working for a long time standing.^13^,^14^

In our study spontaneous abortion was seen two times more in spousal violence exposed women compared to others. Similar to our result in a recent national study it was revealed that women who experienced physical violence were 2.5 times more (OR =2.47, CI:1.37-4.84) experienced miscarriage who did not expose physical violence.^15^ Spontaneous abortion was 1.4 to 1.8 times more in spousal violence exposed women.^16^,^17^ Direct effect of spousal violence is mechanical trauma which ends with spontaneous abortion.^18^ Moreover indirectly women exposed to spousal violence have lower health and social status^19^ and they do not take ANC or postpone.^10^

It is known that women who do not use ANC have more risk of death, delivering low weight babies and more likely to lose their babies in prenatal period.^20^ Our study reveals that spontaneous abortion is seen 2.1 times more in women who do not use ANC within the first 14 weeks. High risk pregnancies could be determined by ANC and their follow up can be made properly so the pregnancy complications can be decreased. Furthermore, adequate information about “signs of danger in pregnancy” can be given to women who use ANC. It was reported that educational status of women and spouse, household income, employment status of women and socio-cultural factors affect the use of ANC.^21^

The strength of this study is inclusion of women with spontaneous abortion within two weeks to minimize the possible mistakes arising from forgetting. Another strength is examining the relation of spontaneous abortions with social and cultural factors. The main limitation of this study was enrollment of only clinically recognized pregnancies ending with spontaneous abortions, so our result may not be generalized for all of spontaneous abortions.

In conclusion, five years or less educational level, employment of women, non-using of ANC during the early period of gestation and spousal violence during pregnancy have been identified as the risk factors for spontaneous abortions. In this context, pregnant women with these risk factors should be followed up more frequently and in a more qualified way.
